# Supplementary material for: Contrasting segregation patterns among endogenous retroviruses across the koala population
Source: Commun Biol. 2024 Mar 21;7:350. doi: 10.1038/s42003-024-06049-0 (PMC10957985; doi:10.1038/s42003-024-06049-0)
Supplement: Supplementary file 2 — Supplementary Information [file 42003_2024_6049_MOESM2_ESM.pdf]

Supplementary information for:

Contrasting segregation patterns among endogenous retroviruses across the koala population

Mette Lillie[1,2*], Mats Pettersson[1], and Patric Jern[1*]

[1]*Science for Life Laboratory, Department of Medical Biochemistry and Microbiology, Uppsala University, SE-751 23, Uppsala, Sweden*

[2]*Department of Ecology and Genetics, Animal Ecology, Uppsala University, SE-752 36, Uppsala, Sweden*

*Correspondence:

Mette Lillie Mette.Lillie@ebc.uu.se

Patric Jern Patric.Jern@imbim.uu.se

This PDF file includes:

Supplementary Fig. 1 – 3

Supplementary Table 1 – 2

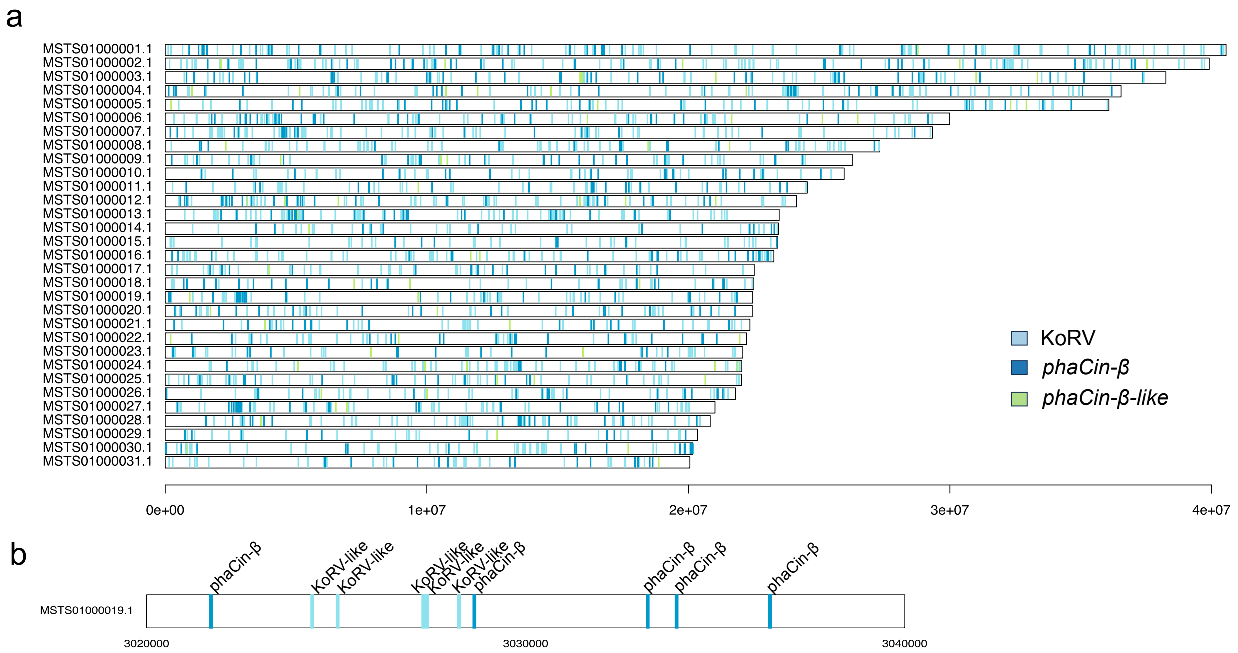

Supplementary Fig. 1 | Population-wide ERV locations along chromosomes. a, Positions of mapped ERVs along the largest koala genome scaffolds (assembled lengths >20 MB) with indicated KoRV, *phaCin-β* and *phaCin-β-like* loci. **b,** Example region on koala scaffold MSTS01000019.1:3,020,000-3,040,000 where 10 loci corresponding to *phaCin-β* (n=5) and KoRV (n=5) were identified across the koala population.

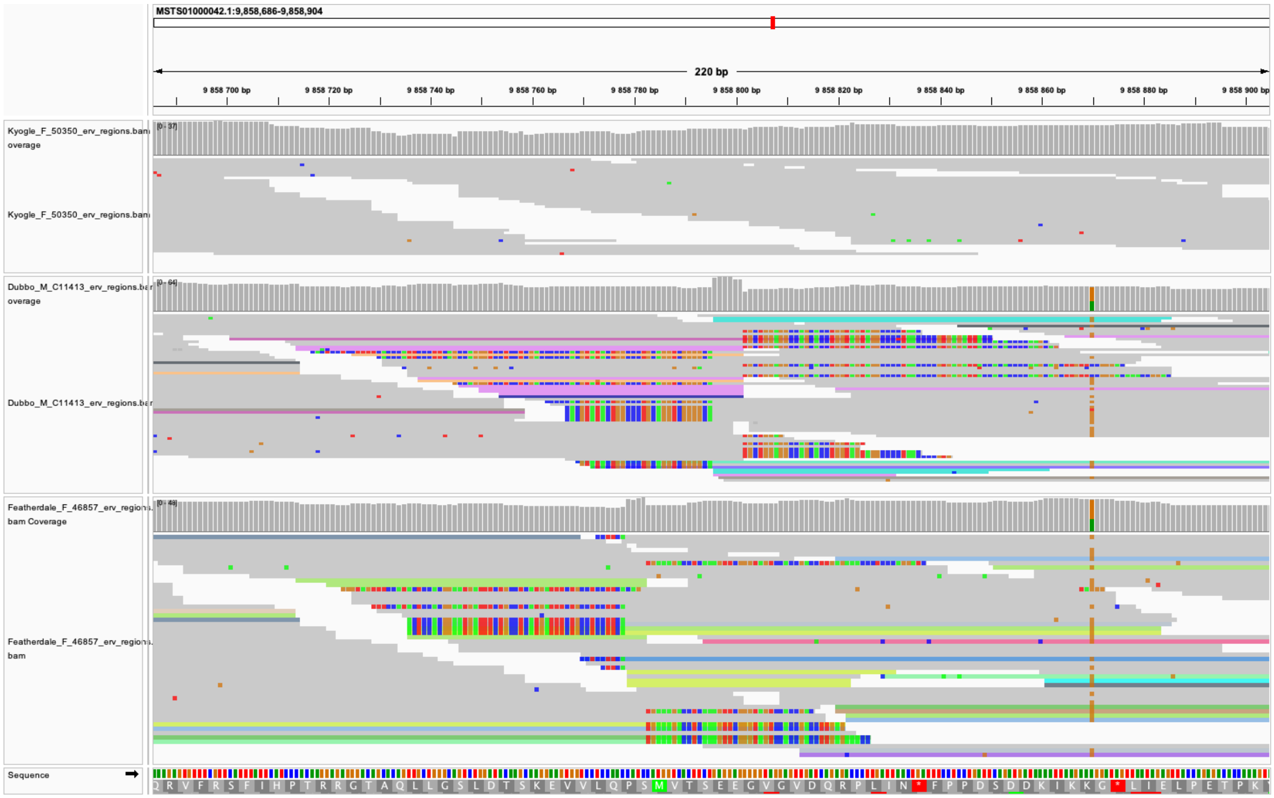

Supplementary Fig 2. | ERV locus confirmation. IGV view of koala genomic region MSTS01000042.1:9,858,686-9,858,904, showing ERV loci identified within close genomic proximity in the koala population. The top panel (Kyogle_F_50350) shows no integrations in this range, the middle panel (Dubbo_M_C11413) indicates a *phaCin-β* integration (position 9,858,795, with a 6 bp target site duplication), and the bottom panel (Featherdale_F_46857) has a KoRV integration (position 9,858,778, with a 4 bp target site duplication).

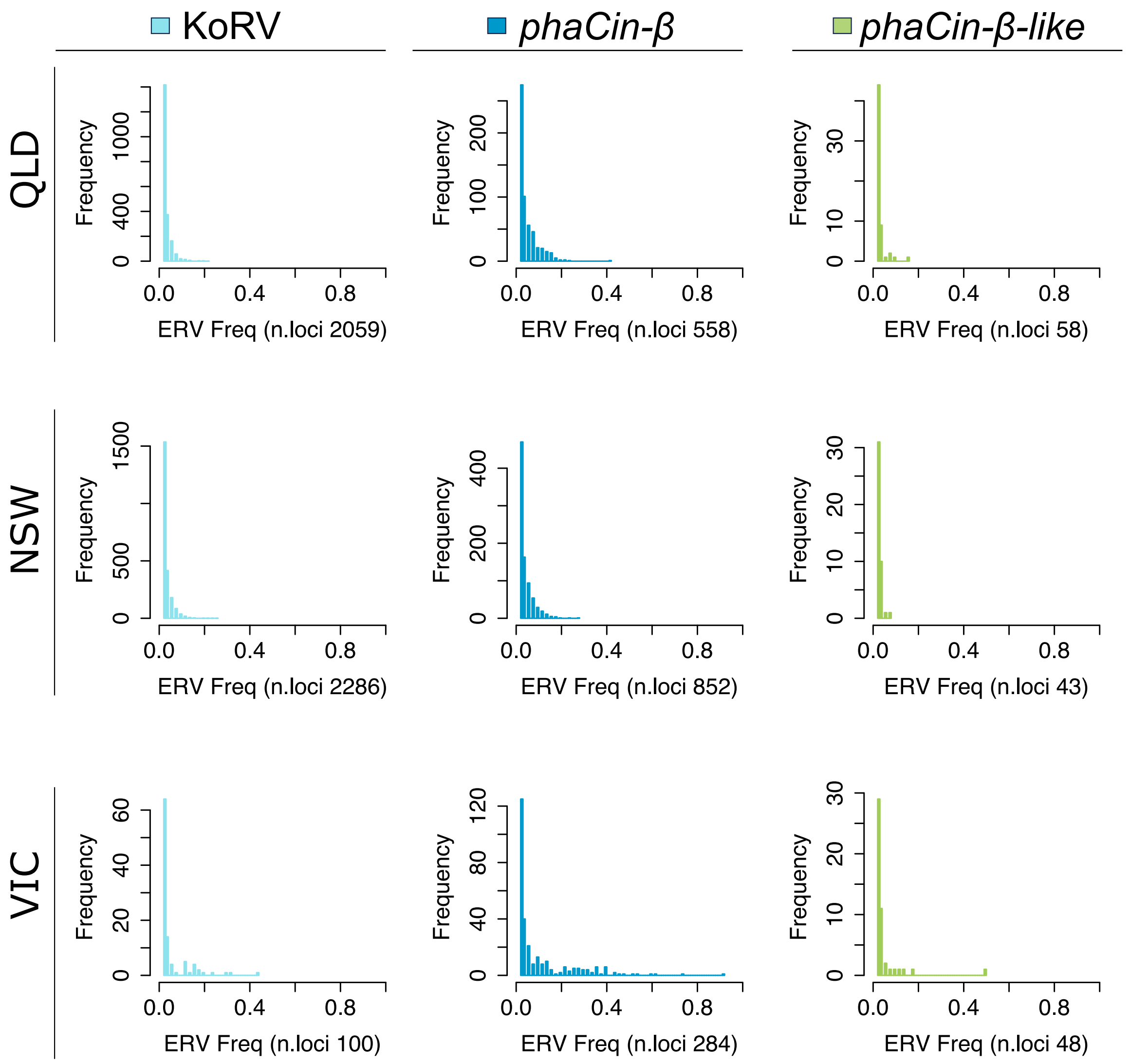

Supplementary Fig. 3 | Histogram of ERV frequencies across Australian states. Queensland (QLD; upper row), New South Wales (NSW; middle row) and Victoria (VIC; lower row) for the three ERV lineages KoRV (left column), *phaCin-β* (middle column) and *phaCin-β-like* (right column). From each state, 50 individuals were randomly sampled, private ERVs to the state identified, and the frequencies of these plotted as histograms (number of private ERVs for each state and ERV lineage indicated by "n.loci"). Similar private ERV frequency patterns can be seen in QLD and NSW for both KoRV and *phaCin-β*, however frequencies in VIC show a contrasting pattern, with intermediate to high frequencies in the state.

Supplementary Table 1 | Sample locations and number of included samples (N) in the koala population ERV screening.

Location (Amazon Web Service Folder Name)	Included sampling sites	N
QLD Fraser Coast	Fraser Coast	10
QLD South Burnett	South Burnett	10
QLD Sunshine Coast	Sunshine coast and Noosa	11
QLD Moreton Bay Region	Moreton Bay	16
QLD Toowoomba	Toowoomba	12
QLD Gold Coast	Gold Coast	12
NSW Kyogle	Kyogle and Northern Rivers	20
NSW Byron	Byron Byron bay and Northern Rivers	13
NSW Lismore	Lismore and Northern Rivers	27
NSW Gunnedah	Gunnedah and Liverpool Plains	26
NSW Port Macquarie	Port Macquarie	13
NSW Port Stephens	Port Stephens	10
NSW Greater Blue Mountains	Blue Mountains	13
NSW Campbelltown	Campbelltown and SW Sydney	20
NSW Wollondilly	STHD	12
NSW Narrandera	Narrandera	17
NSW Monaro	Monaro	20
VIC Moira	Murray river	12
VIC East Gippsland	Gelantipy and Mallacoota	18
VIC South Gippsland	Gippsland and SHGLD	20

Supplementary Table 2 | Homologous *phaCin-β* loci structural variations. Each *phaCin-β* locus is characterized as either pre-integration site (0), provirus (1) or solo-LTR (2) in homologous koala reference assembly (Bilbo) and South Australian assembly (SA) regions.

Locus Bilbo	Locus SA	Bilbo	SA
NW_018343952.1:16776779-16784633	JAOEJA010000741.1:3693979-3700585	1	1
NW_018343953.1:25191880-25191879	JAOEJA010000383.1:15367455-15367785	0	2
NW_018343953.1:3488650	JAOEJA010000383.1:37095927-37101650	0	1
NW_018343954.1:1113280	JAOEJA010001327.1:16494524-16502992	0	1
NW_018343956.1:10725171-10725519	JAOEJA010000643.1:166112233-166112582	2	2
NW_018343956.1:31726436	JAOEJA010000643.1:187134225-187139869	0	1
NW_018343957.1:19834202-19842624	JAOEJA010000514.1:127320655-127329107	1	1
NW_018343960.1:17656158	JAOEJA010000073.1:8568964-8577431	0	1
NW_018343961.1:21305097-21312174	JAOEJA010000656.1:72156087	1	0
NW_018343962.1:16321441	JAOEJA010001159.1:68645483-68653953	0	1
NW_018343962.1:19529052	JAOEJA010001159.1:71869310-71877720	0	1
NW_018343968.1:2177802	JAOEJA010000518.1:69672434-69680900	0	1
NW_018343969.1:1487319	JAOEJA010000885.1:1040756-1046403	0	1
NW_018343970.1:10909918-10918301	JAOEJA010000399.1:21894572-21903025	1	1
NW_018343970.1:2821102	JAOEJA010000399.1:13785669-13794141	0	1
NW_018343971.1:7508696-7517168	JAOEJA010000115.1:48719805	1	0
NW_018343972.1:17325482-17333901	JAOEJA010000594.1:17459762-17468193	1	1
NW_018343972.1:19061308	JAOEJA010000594.1:19196101-19204562	0	1
NW_018343977.1:8895922-8896230	JAOEJA010000643.1:131893467-131899831	2	1
NW_018343978.1:1004264-1004615	JAOEJA010000937.1:38830715-38831067	2	2
NW_018343978.1:17507321-17515793	JAOEJA010000937.1:22311387-22311381	1	0
NW_018343979.1:14125670-14134137	JAOEJA010000643.1:107090776-107090770	1	0
NW_018343981.1:16882197-16890704	JAOEJA010000824.1:18052340-18052333	1	0
NW_018343981.1:20154753	JAOEJA010000824.1:21320126-21320474	0	2
NW_018343981.1:913805-922217	JAOEJA010000097.1:7443712-7452168	1	1
NW_018343986.1:17531244-17539561	JAOEJA010000701.1:16720294-16720287	1	0
NW_018343987.1:24805-33210	JAOEJA010000073.1:88742554-88751006	1	1
NW_018343990.1:17700228-17707162	JAOEJA010000575.1:15013365-15020311	1	1
NW_018343991.1:9188833	JAOEJA010000639.1:21949905-21958377	0	1
NW_018343992.1:15851813	JAOEJA010000399.1:60731282-60739766	0	1
NW_018343998.1:13200036	JAOEJA010000936.1:26338724-26347182	0	1
NW_018344015.1:11021055	JAOEJA010000594.1:33520830-33529288	0	1
NW_018344016.1:11645789-11654227	JAOEJA010000185.1:57612826-57613147	1	2
NW_018344023.1:1970208-1970556	JAOEJA010000042.1:8506053	2	0
NW_018344027.1:5576604-5585072	JAOEJA010001159.1:90961322	1	0
NW_018344028.1:10202984-10211399	JAOEJA010000932.1:10200956-10209393	1	1
NW_018344031.1:1267997-1276433	JAOEJA010000468.1:42278665-42287131	1	1
NW_018344031.1:4359211	JAOEJA010000468.1:39197815-39206277	0	1
NW_018344032.1:61864	JAOEJA010000795.1:33319-38959	0	1

Continued.

Locus Bilbo	Locus SA	Bilbo	SA
NW_018344034.1:1625624	JAOEJA010000922.1:23659367-23665004	0	1
NW_018344035.1:3747592	JAOEJA010000825.1:40756735-40765207	0	1
NW_018344036.1:5232965-5241393	JAOEJA010000643.1:24211526-24219993	1	1
NW_018344038.1:2683935-2684249	JAOEJA010000073.1:125781336-125781651	2	2
NW_018344038.1:5078926-5085302	JAOEJA010000073.1:128190050-128196418	1	1
NW_018344038.1:6202006-6202340	JAOEJA010000073.1:129318884-129319219	2	2
NW_018344042.1:4319930-4328398	JAOEJA010000659.1:6771828	1	0
NW_018344043.1:6379136	JAOEJA010000112.1:6159309-6167774	0	1
NW_018344044.1:9997139	JAOEJA010000548.1:1081107-1089573	0	1
NW_018344046.1:1636918-1645388	JAOEJA010001082.1:40532856	1	0
NW_018344050.1:2061073-2061422	JAOEJA010000656.1:50143272-50143623	2	2
NW_018344052.1:5639248-5639578	JAOEJA010000944.1:5007950	2	0
NW_018344055.1:3287799	JAOEJA010000514.1:10616371-10624381	0	1
NW_018344060.1:1883262-1891724	JAOEJA010000050.1:49846749	1	0
NW_018344064.1:5611633-5611961	AOEJA010000001.1:6481120-6481449	2	2
NW_018344073.1:88015	JAOEJA010000001.1:10688970-10697435	0	1
NW_018344077.1:413575-422011	JAOEJA010000825.1:8970417-8978909	1	1
NW_018344079.1:2092946-2101182	JAOEJA010000643.1:79752382-79760825	1	1
NW_018344081.1:5419770	JAOEJA010000660.1:7293343-7301812	0	1
NW_018344088.1:306475	JAOEJA010000399.1:2812629-2821097	0	1
NW_018344090.1:7142871	JAOEJA010000077.1:7243472-7251935	0	1
NW_018344092.1:5237814-5246236	JAOEJA010000937.1:45088459	1	0
NW_018344094.1:6448134	JAOEJA010000655.1:13805733-13814250	0	1
NW_018344101.1:3685364-3685714	JAOEJA010000828.1:3826629-3826978	2	2
NW_018344115.1:5979649	JAOEJA010000383.1:50945951-50954417	0	1
NW_018344118.1:470197-470545	JAOEJA010000884.1:24680041-24680389	2	2
NW_018344126.1:3147639	JAOEJA010001090.1:23275883-23284345	0	1
NW_018344136.1:2866032	JAOEJA010000936.1:19877661-19886129	0	1
NW_018344141.1:2337383	JAOEJA010001160.1:2328151-2336620	0	1
NW_018344141.1:3743958-3744286	JAOEJA010001160.1:3743056-3743385	2	2
NW_018344142.1:3232771	JAOEJA010000185.1:3362695-3371163	0	1
NW_018344150.1:5531959	JAOEJA010000279.1:4281625-4290094	0	1
NW_018344160.1:4450675	JAOEJA010000824.1:1026382-1034849	0	1
NW_018344170.1:3741391	JAOEJA010000518.1:91250655-91256307	0	1
NW_018344175.1:3999554	JAOEJA010000073.1:84691974-84692323	0	2
NW_018344182.1:3982440-3982767	JAOEJA010001333.1:6136880	2	0
NW_018344205.1:202785	JAOEJA010001284.1:3547825-3556289	0	1
NW_018344206.1:2610228-2610577	JAOEJA010000932.1:15168892-15169242	2	2
NW_018344231.1:3086220	JAOEJA010000073.1:57018669-57027136	0	1
NW_018344236.1:145019-145341	JAOEJA010000279.1:14361675-14370112	2	1
NW_018344242.1:2231815-2232144	JAOEJA010001095.1:16710732-16711062	2	2
NW_018344243.1:692853-701323	JAOEJA010000050.1:75477209	1	0
NW_018344260.1:682177	JAOEJA010000548.1:26237512-26245975	0	1
NW_018344287.1:523019-523013	JAOEJA010000531.1:589831-598299	0	1
NW_018344297.1:1640839-1649312	JAOEJA010000941.1:751658-760157	1	1

Continued.

Locus Bilbo	Locus SA	Bilbo	SA
NW_018344347.1:460174	JAOEJA010000279.1:562112-570579	0	1
NW_018344443.1:40288-40637	JAOEJA010000270.1:104856-105205	2	2
NW_018344501.1:34883-43828	JAOEJA010000138.1:475230-475698	1	2
NW_018344659.1:26558	JAOEJA010000275.1:88793-97260	0	1
NW_018345530.1:12850	JAOEJA010000497.1:52250-60744	0	1
NW_018345562.1:10256	JAOEJA010000312.1:335038-343506	0	1
NW_018345562.1:6426	JAOEJA010001139.1:68502-76969	0	1
NA	JAOEJA010000872.1:5066-13211	NA	1
NA	JAOEJA010001074.1:18549-27019	NA	1
NA	JAOEJA010000062.1:43644-52104	NA	1
NA	JAOEJA010000192.1:0-8461	NA	1
NA	JAOEJA010000262.1:14610-20253	NA	1
NA	JAOEJA010000604.1:8678-17053	NA	1
NA	JAOEJA010000871.1:5150-13598	NA	1
