# Supplementary material for: Contrasting segregation patterns among endogenous retroviruses across the koala population
Source: Commun Biol. 2024 Mar 21;7:350. doi: 10.1038/s42003-024-06049-0 (PMC10957985; doi:10.1038/s42003-024-06049-0)
Supplement: Supplementary file 3 — Reporting summary [file 42003_2024_6049_MOESM3_ESM.pdf]

nature portfolio

Corresponding author(s): Lillie, M
Jern, P

Last updated by author(s): Nov 14, 2023

Reporting Summary

Nature Portfolio wishes to improve the reproducibility of the work that we publish. This form provides structure for consistency and transparency in reporting. For further information on Nature Portfolio policies, see our Editorial Policies and the Editorial Policy Checklist.

Statistics

For all statistical analyses, confirm that the following items are present in the figure legend, table legend, main text, or Methods section.

n/a	Confirmed	
☐	☒	The exact sample size (*n*) for each experimental group/condition, given as a discrete number and unit of measurement
☒	☐	A statement on whether measurements were taken from distinct samples or whether the same sample was measured repeatedly
☒	☐	The statistical test(s) used AND whether they are one- or two-sided<br>*Only common tests should be described solely by name; describe more complex techniques in the Methods section.*
☒	☐	A description of all covariates tested
☒	☐	A description of any assumptions or corrections, such as tests of normality and adjustment for multiple comparisons
☐	☒	A full description of the statistical parameters including central tendency (e.g. means) or other basic estimates (e.g. regression coefficient) AND variation (e.g. standard deviation) or associated estimates of uncertainty (e.g. confidence intervals)
☒	☐	For null hypothesis testing, the test statistic (e.g. *F*, *t*, *r*) with confidence intervals, effect sizes, degrees of freedom and *P* value noted<br>*Give P values as exact values whenever suitable.*
☒	☐	For Bayesian analysis, information on the choice of priors and Markov chain Monte Carlo settings
☒	☐	For hierarchical and complex designs, identification of the appropriate level for tests and full reporting of outcomes
☒	☐	Estimates of effect sizes (e.g. Cohen's *d*, Pearson's *r*), indicating how they were calculated

Our web collection on statistics for biologists contains articles on many of the points above.

Software and code

Policy information about availability of computer code

Data collection	Open access data available from Amazon Web Services Open Data platform (https://registry.opendata.aws/australasian-genomics/) using the AWS Command Line Interface.
Data analysis	ERV detection and segregation: RetroTector v1.0 ,RetroSeq https://github.com/tk2/RetroSeq (Accessed Feb. 18, 2020), DELLY v0.7.7, Lumpy v0.3.0, BLAT v36, BCFtools v1.14, R v4.1.3, and<br>R packages: GenomicRanges v1.46.1, intansv v1.34.0, VariantAnnotation v1.40.0, data.table v1.14.2, pheatmap v1.0.12, ggplot2 v3.4.1, regioneR v1.26.1, StructuralVariantAnnotation v1.10.1, geodist v0.0.8, dplyr v1.1.1<br>Custom R scripts for analysis and RetroTector software is available via https://github.com/PatricJernLab/

For manuscripts utilizing custom algorithms or software that are central to the research but not yet described in published literature, software must be made available to editors and reviewers. We strongly encourage code deposition in a community repository (e.g. GitHub). See the Nature Portfolio guidelines for submitting code & software for further information.

Data

Policy information about availability of data

All manuscripts must include a data availability statement. This statement should provide the following information, where applicable:

- Accession codes, unique identifiers, or web links for publicly available datasets
- A description of any restrictions on data availability
- For clinical datasets or third party data, please ensure that the statement adheres to our policy

The reference koala assembly is available at Genbank: GCA_002099425.1 [https://www.ncbi.nlm.nih.gov/datasets/genome/GCF_002099425.1/]. The South Australian koala assembly is available at Genbank: GCA_030178435.1 [https://www.ncbi.nlm.nih.gov/datasets/genome/GCA_03017835.1/]. Whole genome sequencing data is available from Amazon Web Services Open Data platform (https://registry.opendata.aws/australasian-genomics/)

Research involving human participants, their data, or biological material

Policy information about studies with human participants or human data. See also policy information about sex, gender (identity/presentation), and sexual orientation and race, ethnicity and racism.

Reporting on sex and gender	Study did not involve human participants
Reporting on race, ethnicity, or other socially relevant groupings	Study did not involve human participants
Population characteristics	Study did not involve human participants
Recruitment	Study did not involve human participants
Ethics oversight	Study did not involve human participants

Note that full information on the approval of the study protocol must also be provided in the manuscript.

Field-specific reporting

Please select the one below that is the best fit for your research. If you are not sure, read the appropriate sections before making your selection.

☐ Life sciences ☐ Behavioural & social sciences ☒ Ecological, evolutionary & environmental sciences

For a reference copy of the document with all sections, see nature.com/documents/nr-reporting-summary-flat.pdf

Ecological, evolutionary & environmental sciences study design

All studies must disclose on these points even when the disclosure is negative.

Study description	We investigate geographic polymorphism focusing on three distinct ERV lineages from recent expansions in koala , using the whole-genome sequencing dataset of 430 koalas produced by the Koala Genome Survey.
Research sample	Whole-genome sequencing dataset of 430 koalas produced by the Koala Genome Survey, including mainly tissue (ear biopsy) samples, as well as blood samples. We did not collect these samples or generate this sequencing data; rather it is an open data source available via AWS.
Sampling strategy	We did not collect these samples nor generate the sequencing data; rather it is an open data source available via AWS. Details are available in Hogg et al 2023 "Koala Genome Survey: An Open Data Resource to Improve Conservation Planning" in Genes 14.546
Data collection	We did not collect these samples nor generate the sequencing data; rather it is an open data source available via AWS. Details are available in Hogg et al 2023 "Koala Genome Survey: An Open Data Resource to Improve Conservation Planning" in Genes 14.546
Timing and spatial scale	We did not collect these samples nor generate the sequencing data; rather it is an open data source available via AWS. Details are available in Hogg et al 2023 "Koala Genome Survey: An Open Data Resource to Improve Conservation Planning" in Genes 14.546
Data exclusions	No data was excluded from analysis, except where stated that the sample set was filtered for certain population comparisons where only populations with 10 or more samples were included in figures
Reproducibility	not applicable
Randomization	not applicable

Blinding | not applicable

Did the study involve field work? ☐ Yes ☒ No

Reporting for specific materials, systems and methods

We require information from authors about some types of materials, experimental systems and methods used in many studies. Here, indicate whether each material, system or method listed is relevant to your study. If you are not sure if a list item applies to your research, read the appropriate section before selecting a response.

Materials & experimental systems

n/a	Involved in the study	
☒	☐	Antibodies
☒	☐	Eukaryotic cell lines
☒	☐	Palaeontology and archaeology
☒	☐	Animals and other organisms
☒	☐	Clinical data
☒	☐	Dual use research of concern
☒	☐	Plants

Methods

n/a	Involved in the study	
☒	☐	ChIP-seq
☒	☐	Flow cytometry
☒	☐	MRI-based neuroimaging

Plants

Seed stocks | *Report on the source of all seed stocks or other plant material used. If applicable, state the seed stock centre and catalogue number. If plant specimens were collected from the field, describe the collection location, date and sampling procedures.*

Novel plant genotypes | *Describe the methods by which all novel plant genotypes were produced. This includes those generated by transgenic approaches, gene editing, chemical/radiation-based mutagenesis and hybridization. For transgenic lines, describe the transformation method, the number of independent lines analyzed and the generation upon which experiments were performed. For gene-edited lines, describe the editor used, the endogenous sequence targeted for editing, the targeting guide RNA sequence (if applicable) and how the editor was applied.*

Authentication | *Describe any authentication procedures for each seed stock used or novel genotype generated. Describe any experiments used to assess the effect of a mutation and, where applicable, how potential secondary effects (e.g. second site T-DNA insertions, mosiacism, off-target gene editing) were examined.*
